# Conflict of Interest Policies at Medical Schools and Teaching Hospitals: A Systematic Review of Cross-sectional Studies

**DOI:** 10.34172/ijhpm.2021.12

**Published:** 2021-03-03

**Authors:** Alice Fabbri, Kristine Rasmussen Hone, Asbjørn Hróbjartsson, Andreas Lundh

**Affiliations:** ^1^Centre for Evidence-Based Medicine Odense (CEBMO), University of Southern Denmark and Odense University Hospital, Odense, Denmark.; ^2^Tobacco Control Research Group, Department for Health, University of Bath, Bath, United Kingdom.; ^3^Centre for Evidence-Based Medicine Odense (CEBMO) and Cochrane Denmark, Department of Clinical Research, University of Southern Denmark, Odense, Denmark.; ^4^Open Patient data Explorative Network (OPEN), Odense University Hospital, Odense, Denmark.; ^5^Department of Infectious Diseases, Hvidovre Hospital, Hvidovre, Denmark.

**Keywords:** Conflict of Interest, Policies, Industry, Medical Schools, Teaching Hospitals

## Abstract

**Background:** This systematic review aims to estimate the proportion of medical schools and teaching hospitals with conflicts of interest (COI) policies for health research and education, to describe the provisions included in the policies and their impact on research outputs and educational quality or content.

**Methods:** Experimental and observational studies reporting at least one of the above mentioned aims were included irrespective of language, publication type or geographical setting. MEDLINE, Scopus, Embase and the Cochrane Methodology Register were searched from inception to March 2020. Methodological study quality was assessed using an amended version of the Joanna Briggs Institute’s checklist for prevalence studies.

**Results:** Twenty-two cross-sectional studies were included; all were conducted in high-income countries. Of these, 20 studies estimated the prevalence of COI policies, which ranged from 5% to 100% (median: 85%). Twenty studies assessed the provisions included in COI policies with different assessment methods. Of these, nine analysed the strength of the content of medical schools’ COI policies using various assessment tools that looked at a range of policy domains. The mean standardised summary score of policy strength ranged from 2% to 73% (median: 30%), with a low score indicating a weak policy. North American institutions more frequently had COI policies and their content was rated as stronger than policies from European institutions. None of the included studies assessed the impact of COI policies on research outputs or educational quality or content.

**Conclusion:** Prevalence of COI policies at medical schools and teaching hospitals varied greatly in high-income countries. No studies estimated the prevalence of policies in low to middle-income countries. The content of COI policies varied widely and while most European institutions ranked poorly, in North America more medical schools had strong policies. No studies were identified on impact of COI policies on research outputs and educational quality or content.

## Background

 In the past decades, academics’ financial ties with commercial companies have been highly debated. Studies suggest that such relationships can introduce biases in research and education.^1^ For example, both industry funding of medical research and financial relationships between academic researchers and commercial companies are associated with conclusions favourable to the companies’ products.^2-5^ Moreover, industry funding can impose constraints on publication rights and access to the raw data which can potentially lead to selective reporting of favourable outcomes and withholding of unfavourable results.^6,7^ Conflicts of interest (COI) can also influence medical education.^1^

 These concerns have led some medical schools to implement institutional policies to regulate interactions with commercial entities in order to protect the integrity of the research and educational process. Some studies conducted in North America have examined how medical schools vary in their implementation of COI policies.^8-11^ More recently, analyses of COI policies have been conducted in some European countries.^12,13^

 COI policies likely have regional differences and have changed over time. However, to our knowledge no previous systematic reviews have comprehensively identified and analysed studies on COI policies of medical schools and teaching hospitals to protect the research and educational environment from commercial influences.

 The objectives of this review are:

1. To describe the proportion of medical schools and teaching hospitals with COI policies for health research and education.
2. For the medical schools and teaching hospitals with COI policies:
  - To describe the provisions included in the policies and to whom they apply.
  - To describe the impact of COI policies on research outputs, educational quality or content.

## Methods

###  Protocol, Search Strategy, and Study Selection

 The protocol was published in PROSPERO (CRD42020197500). We searched the following databases (from inception to March 9, 2020): Ovid MEDLINE, Scopus, Ovid Embase, and the Cochrane Methodology Register. The search strategy was developed in consultation with an information specialist (Supplementary file 1). We also searched reference lists of included studies and Web of Science for publications that cited any of the included studies. For included studies available in unpublished form (eg, pre-prints), in October 2020 we checked whether the study had been published and updated the information we had previously extracted.

###  Eligibility Criteria 

 We included experimental and observational studies investigating COI policies of medical schools or teaching hospitals. For the purpose of this study, we considered COI policies any document or regulation on interactions with industry both at the individual level (eg, researchers, medical students) and institutional level. We focused on COI policies to protect research and education and did not restrict to policies related to the pharmaceutical industry. We included any study with one or more of the following aims:

- Estimating the prevalence of COI policies.
- Describing the content and scope of COI policies (eg, which activities should be disclosed, which activities are prohibited or constrained).
- Estimating the impact of COI policies on research output (eg, number of trials conducted after the implementation of the policy), educational quality or content. By impact we mean an objective measure, not a subjective one (eg, opinions of staff about the policy).

We excluded editorials, letters, commentaries, descriptive case studies, systematic and narrative reviews; studies on schools not in the medical sciences field (eg, engineering); studies that included different types of institutions, without separate data reported for medical schools or teaching hospitals. We excluded studies solely focusing on policies to protect clinical practice and studies that focused on one single element of a COI policy (eg, disclosure only, gifts only) rather than the breadth of policies in medical schools or teaching hospitals. We did not exclude studies based on language, publication date, or study setting.

###  Study Inclusion

 One assessor (AF) screened the titles and abstracts of retrieved records. Two assessors (AF, KRH) independently assessed the full texts of the remaining records for inclusion. Discrepancies were resolved by discussion. If consensus could not be reached, a third assessor adjudicated (AL).

###  Data Collection 

 For each study we collected information on: general study information (author, publication year, funding source and authors’ financial COI), study design, setting, and population, study period, methods of data collection, and outcomes (any quantitative data was extracted on the above mentioned aims: prevalence of COI policies, content, scope and impact of the policies).

 One assessor (AF) extracted information from records and a second assessor (KRH) checked the accuracy and completeness of extracted information. Discrepancies were resolved by discussion. When information reported in included records was unclear or if relevant data was missing, we contacted the study authors for clarifications or unpublished data. We contacted the authors of 13 studies: 11 replied and nine of these supplied the requested information.

###  Methodological Quality Assessment 

 We used an adapted version of the Checklist for Studies Reporting Prevalence Data developed by Joanna Briggs Institute to assess methodological quality.^14^ The original checklist assesses the quality of a study across nine items. We excluded items related to statistical issues and reporting quality and only included five items strictly related to methodological quality: sample frame, methods used to recruit participants, methods for identification of outcomes, measurement of outcomes and missing data. We changed the possible answers for each item from yes, no, unclear, or not applicable to high quality, low quality, unclear, or not applicable to reflect methodological quality (ie, ‘yes’ for appropriate sample frame was changed to ‘high quality’ for sample frame). We developed guidance for how to assess each item (Supplementary file 2) drawing inspiration from a previous systematic review where the guide for item assessment had been amended to reflect the focus on a policy issue rather than a clinical condition.^15^ Studies with high quality in all domains were assessed as overall high quality and other studies as overall low quality.

###  Analysis

 We undertook an initial descriptive analysis of the included studies, presenting their characteristics, settings, and populations. We grouped studies based on which of our study aims they addressed. Any commonalities between studies was synthesised and discussed.

####  Prevalence of Policies

 For studies estimating prevalence of COI policies, we calculated confidence intervals for individual studies using the Clopper-Pearson method.^16^ Since the studies on prevalence were very heterogeneous, for example in relation to study setting, methodology and study period, no summary estimate was calculated. However, we undertook meta-analysis (random effects meta-analysis using the DerSimonian-Laird estimate of single proportions with prevalence estimates that had been transformed using the Freeman-Tukey double arcsine transformation)^17,18^ in order to assess heterogeneity using I^2^ and explored reasons for heterogeneity in the following prespecified subgroup analyses: setting (North America versus other settings), timing (before versus after median publication year), and methodological quality (high versus low quality). Statistical analyses were conducted in MedCalc 19.6.

####  Content of Policies

 For studies investigating content of COI policies, we grouped the studies that addressed this question in four different categories based on the assessment methods and the target institutions: (1) studies assessing the strength of the content of COI policies of medical schools using various assessment tools; (2) studies assessing the strength of the content of COI policies of teaching hospitals; (3) studies examining the content of COI policies without assessing their strength, and (4) studies examining the content of medical school and hospital-based Institutional Review Boards’ (IRBs’) COI policies. Moreover, for studies that used assessment tools (eg, checklists, scales) to analyse the strength of the content of COI policies (ie, category 1), we coded the items included in each tool and grouped them into different domains to synthesize their commonalities. In order to compare the results of these studies, for tools including a summary score (ie, scales), we also standardised the overall scores by dividing the mean score with the maximum score possible for a given tool and reported this as percentages. We ensured directionality of scales (ie, that high scores indicated high strength of policy). In our main analysis we included both institutions with a COI policy and institutions without a COI policy in the denominator for calculating mean standardised summary scores (ie, institutions without a COI policy will automatically receive a score of 0). In a sensitivity analysis, we restricted our analysis to institutions with available COI policies.

####  Association Between Policies and Research Output or Educational Quality or Content 

 We planned to calculate pooled estimates for the association between COI polices and research output, or educational quality or content, but no relevant studies were identified.

## Results

###  Description of Included Studies

**Figure 1:**
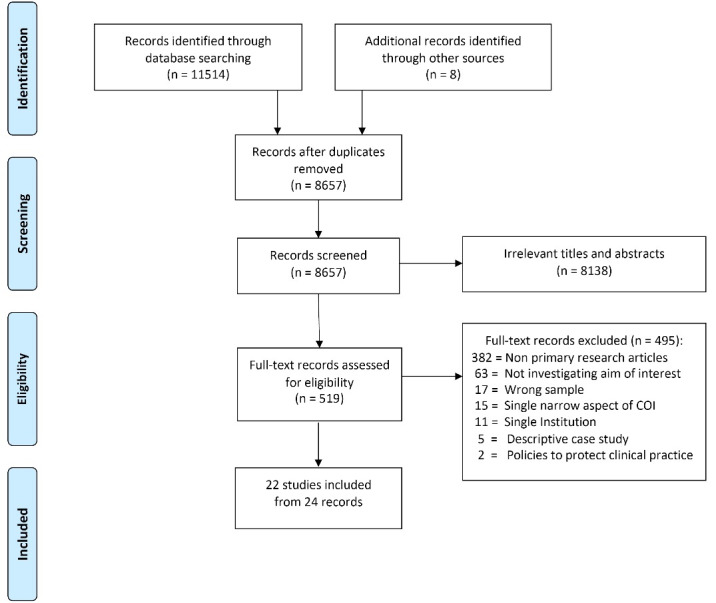
PRISMA Flow Diagram of Included Studies. Abbreviations: COI, conflicts of interest; PRISMA, Preferred Reporting Items for Systematic Reviews and Meta-Analyses.

 We identified 8657 records for screening and included 22 studies (from 24 records) (Figure 1). The most common reason for exclusion was type of publications (not primary research articles, n = 382), followed by aims of interest not investigated (n = 63). For two records, we could not retrieve full text versions despite contacting four medical libraries. None of the records had abstracts, none of the titles suggested that they were original research, and one was only one page long. We therefore judged these records as unlikely to report on primary research.^19,20^ For one study a preprint version was initially included, but a journal publication was also included during the additional search run in October 2020.^12^

Table 1 summarises the characteristics of the included studies. The 22 studies were published between 2000 and 2020 and all were cross-sectional. All the studies were conducted in high-income countries, primarily North America (n = 17). Several studies used data collected from multiple sources, such as policies retrieved from both institutional websites and through contact with University officials. Nine studies were surveys of institutional members and their response rates ranged from 62% to 100% (median 69%).

**Table 1:** Characteristics of 22 Included Cross-sectional Studies

**Study**	**Location of Institutions**	**Period of Data Collection**	**Target Institutions** ^a^	**Aims (This Refers to the Aims of Our Review)**	**Key Data Collection Methods** ^b^	**Response Rate (If Applicable)** ^c^	**Funding Source**	**Author Financial COI (Only Ties With Industries)**
Campbell, 2006	US	2005	100 Medical schools and 15 hospitals that received the most funding from the NIH in 2003	Prevalence and content	Survey of IRBs members	574/854 (67%)	Public	No
Campbell, 2015	US	2014	100 Medical schools and 15 teaching hospitals that received the most funding from the NIH in 2012	Prevalence	Survey of IRB members	593/866 (69%)	Public	No
Carlat, 2016	US	2013 and 2014	Medical schools (n = 158 in 2013; n = 160 in 2014)	Content	Policies identified via email to Deans and conflict of interest officers, search of institutional websites, search of AMSA and IMAP website	n/a	Public and non-profit	1 of 5 authors has industry ties
Chimonas, 2011	US	October 2007-December 2008	125 Medical schools	Content	Survey of Deans and Compliance officers + Policies identified via contacts with Deans and Compliance officers	77/125 (62%)	Public and non-profit	No
Chimonas, 2013	US	July-September 2011	133 Medical schools	Prevalence and content	Policies identified via search of institutional websites, and email and telephone contacts with Deans and compliance officers if no policies were found online	n/a	Public and non-profit	No
Ehringhaus, 2008	US	February-December 2006	125 Medical schools	Prevalence and content	Survey of deans, acting deans, or interim deans	86/125 (69%)	Non-profit	No
Grabitz, 2020	Germany	May-October 2018	38 Medical schools	Prevalence and content	Policies identified via search of institutional websites and email contacts with the Dean’s offices	n/a	No external funding received	No
Guy-Coichard, 2019	France	May-December 2017	32 Teaching hospitals	Prevalence and content	Policies identified via search of institutional websites and mail and email contacts with chief executive officer of the teaching hospital	n/a	Non-profit	No
Kaufman, 2004	Canada	2000-2002	16 Faculties of medicine and their parent universities	Prevalence	Survey of faculties of medicine and parent universities	16/16 (100%)	Public and non-profit	Not reported
Klein, 2018^d^	US	2016	65 Universities training physicians and advanced practice registered nurses	Prevalence	Survey of University officials. Answers were compared with data from policies identified through the websites	20/65 (30%)	Non-profit	No
Lexchin, 2008	Canada	August 2005-May 2006	16 Universities with faculties of medicine, 16 faculties of medicine, and 47 teaching hospitals	Prevalence and content	Policies identified via search of institutional websites and contacts with vice-presidents for research or equivalent	n/a	Public	No
Lieb, 2014	Germany	November 2011-March 2012	36 Medical faculties	Prevalence and content	Survey of student affairs' Deans	30/36 (83%)	Not reported	No
Lo, 2000	US	June-July 2000	10 Medical schools that receive the largest amount of research funding from the NIH	Prevalence and content	Policies identified from institutional websites, telephone or e-mail contact with officials from University offices for research affairs, research administration, contracts and grants, or compliance or IRB	n/a	Public and non-profit	Not reported
Mason, 2011	Australia	October 2009	20 Medical schools	Prevalence and content	Policies identified via written contacts with the Deans	n/a	Not reported	No
Mathieu, 2012	Canada	March-September 2010	16 Universities hosting medical schools	Prevalence and content	Policies identified via search of institutional websites	n/a	Public and non-profit	No
Rochon, 2010^e^	Canada	August 2005-February 2006	16 Medical schools and 47 teaching hospitals as well as their 16 partner universities	Prevalence and content	Policies identified via search of institutional websites and contacts with vice-presidents for research (or equivalent)	n/a	Public	No
Scheffer, 2017	France	May-December 2015	37 Medical faculties	Prevalence and content	Policies identified via search of the institutional websites and email contact with Deans	n/a	No external funding received	No
Shnier, 2013	Canada	July- September 2011	17 Medical schools	Prevalence and content	Policies identified via search of institutional websites and email contacts with the Deans	n/a	No external funding received	No
Sierles, 2005	US	2005	126 Medical schools	Prevalence	Survey of student affairs and educational affairs Deans (mailed survey)	110/126 (87%)	Public and non-profit	1 of 11 authors has industry ties
Weinfurt, 2010	US	May-September 2008	92 Academic medical centres that participated in industry-sponsored phase 3 trials published in *JAMA* or *New England Journal of Medicine* in 2006-2007	Prevalence and content	Survey of officials (multiple individuals at each site could assist in the completion of the survey)	61/92 (66%)	Public	No
Wolf, 2007	US	March 2004-June 2005	124 IRBs from the 121 medical schools that received NIH funding in fiscal year 2003	Prevalence and content	Policies identified via search of institutional websites and contacts with IRB representatives and with administrators of the conflict of interest committees	n/a	Not reported	Not reported
Yeh, 2014	US	2010	121 medical schools	Prevalence and content	Secondary analysis of data on COI policies previously collected by AMSA and IMAP in 2010	n/a	Public and non-profit	No

Abbreviations: AMSA, American Medical Student Association; COI, conflicts of Interest; IMAP, Institute of Medicine as a Profession; IRB, Institutional Review Board; NIH, National Institutes of Health. ^a^We coded the institutions as reported by the authors (eg, medical schools, Faculties of Medicine). This could reflect language or geographical differences. ^b^Some studies used several data collection methods (eg, websites analyses, questionnaires). Only those used to collect the data included in this systematic review are reported. ^c^We reported the response rate only for surveys (namely studies where the authors used questionnaires to ask specific questions and the respondents should then provide answers). Several studies contacted University officials to obtain COI policies whose content was then assessed by the study authors. We did not consider these studies as surveys and therefore did not report the response rate. ^d^Most respondents (n = 17) were Deans of Colleges of Nursing or program directors. After correspondence with the first author, we clarified that all the included institutions are academic colleges of medicine but some also educate advanced practice registered nurses. ^e^Rochon, 2010 included the same institutions as the study by Lexchin, 2008. The difference between the studies is that Lexchin investigated COI policies as they applied to researchers within the institution whereas Rochon investigated policies on institutional financial COI.

###  Methodological Quality of Included Studies

**Figure 2:**
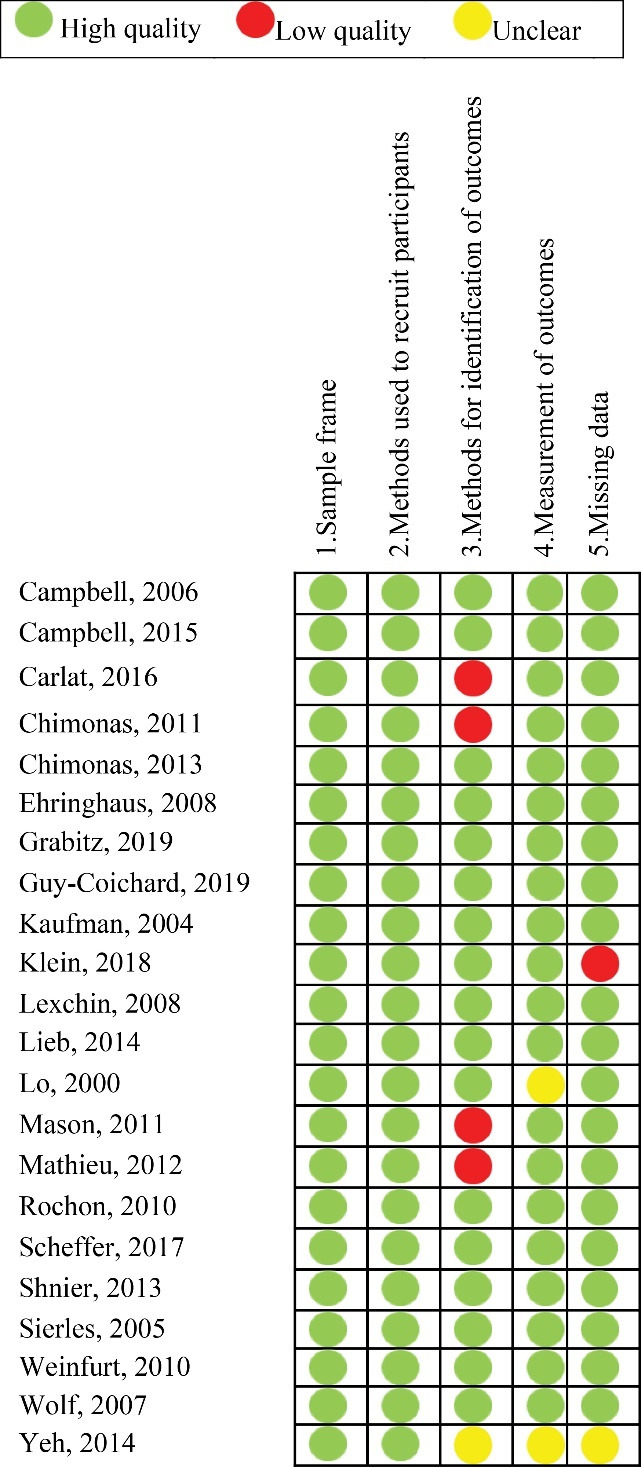
Methodological Quality Assessment of Included Studies.

Figure 2 shows the quality assessment of the included studies. Fifteen of 22 studies were assessed as overall high quality (ie, high quality for all domains). The domain on methods for identification of outcomes was the most frequent domain judged as low quality (n = 4/22).

####  Prevalence of COI Policies 

**Figure 3:**
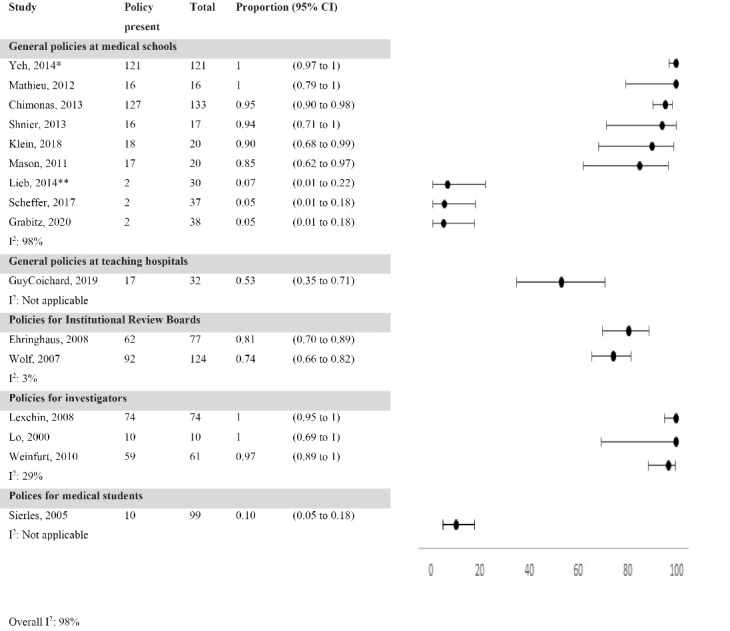
Forest Plot of Prevalence of COI Policies. Abbreviation: COI, conflicts of Interest. *Data provided by the author. **Lieb, 2014 asked about the existence of a general COI policy and/or a policy on interactions between medical students and industry. I^2^: Heterogeneity described by calculating I^2^.

 Twenty studies estimated the prevalence of COI policies with estimates ranging from 5 to 100% (median: 85%). We grouped the studies into categories according to their focus (Figure 3). Nine studies estimated prevalence of general COI policies in medical schools; prevalence 5% to 100% (median: 90%).^8,9,12,13,21-25^ Some of these studies only included policies of medical schools while two clearly stated that they also included COI policies of the parent University.^8,21^ One French study reported that 53% (17/32) of teaching hospitals had a general COI policy.^26^ Two studies estimated prevalence of COI policies for members of medical school-based IRBs in the United States; prevalence 74% and 81%.^27,28^ Three studies estimated prevalence of COI policies for investigators from Universities or teaching hospitals in Canada and the United States; prevalence from 97% to 100%.^11,29,30^ One study reported that 10% (10/99) of US medical schools had COI policies specifically targeting industry-student relationships.^31^

 Finally, four studies were not included in Figure 3 because of their distinct focus: the first study reported that none of 16 Canadian Faculties of Medicine or their parent universities had policies regarding research funding or financial donations from tobacco companies^32^; the second study focused on COI policies solely at the institutional level among 74 Canadian teaching hospitals, medical schools, and their parent Universities; prevalence of 55% (41/74).^33^ The last two studies estimated prevalence of COI policies for US medical school and teaching hospital-based IRBs via a survey of their members; prevalence 46% (263/574) and 63% (311/493).^34,35^ However, these estimates are not comparable to the other two studies on IRBs^27,28^ reported in Figure 3 because prevalence was measured at member level and not at institutional level (ie, some members included in survey were from same institution).

###  Content and Strength of COI Policies

 We grouped the 20 studies that assessed the provisions included in COI policies in four categories based on the assessment methods and the target institutions. Five studies across the four categories reported data on who the policies applied to and all studies found that policies often did not cover all relevant stakeholders (eg, only selected faculty) (Supplementary files 3 and 4). Nine studies assessed the presence of policy enforcement mechanisms such as oversight and sanctions for non compliance and found that they were not consistently mentioned in the included policies (Supplementary files 3 and 4).

####  Strength of the Content of COI Policies of Medical Schools 

 Nine studies analysed the strength of the content of COI policies of medical schools (in two cases also their parent universities) using assessment tools that looked at a range of policy domains.^8-10,12,13,22-24,36^ All assessment tools used a scale approach (ie, individual scores for each item transformed into an overall summary score). Table 2 presents the range of policy domains addressed by the various assessment tools. The number of items in each tool ranged from 7 to 14. We grouped the items into 14 common domains and one ‘other group.’ Seven of the nine studies used the American Medical Student Association (AMSA) scorecard or an adaptation of the scorecard as assessment tools.

 The mean standardised summary score ranged from 2% to 73% (median: 30%). The European studies both had a summary score of 2% while in the North American studies the score ranged from 22% to 73%. The sensitivity analysis, restricting analysis to institutions with policies, increased the strength of policies from European institutions and thereby reduced the gap between European and US institutions, but still important differences were detected (Supplementary file 3, Table S1).

**Table 2:** Characteristics of the 9 Studies That Systematically Assessed the Strength of the Content of COI Policies of Medical Schools Using an Assessment Tool

**Study** ^a^	**Assessment Tool Used**	**No. of Items Included in Assessment Tool **	**Domains Included in Assessment Tools**															**Mean Standardised Summary Score** ^d^
			**Gifts**	**Meals**	**Sales Reps**	**Honoraria**	**Consulting **	**Industry Scholarships**	**Ghostwriting**	**Speakers’ Bureaus**	**Disclosure**	**Samples**	**Attendance of Promotional Events**	**Industry Sponsorship of Educational Events **	**Travel/Off-site Education**	**P&T/Purchasing Committee Membership** ^b^	**Other** ^c^	
Carlat, 2016^e^	Revision of AMSA scorecard	14	Yes	Yes	Yes		Yes	J	Yes	Yes	Yes		Yes	Yes	J		Yes	2013: 73% 2014: 56%
Chimonas, 2011	Coding system designed by authors	11	Yes	Yes	Yes	Yes	Yes	J	Yes	Yes		Yes		Yes	J	Yes		40%
Chimonas, 2013	Coding system designed by authors	12	Yes	Yes	Yes	Yes	Yes	Yes	Yes	Yes		Yes		Yes	Yes	Yes		2008: 27% 2011: 56%
Grabitz, 2020	Authors’ adaptation from AMSA scorecard, Scheffer 2017, Shnier 2013	13	Yes	Yes	Yes	J	Yes	J	Yes	Yes	Yes		Yes	Yes			Yes	2%
Mason, 2011	Authors’ adaptation of AMSA scorecard	7	J	J			Yes			Yes	Yes			Yes	Yes			26%
Mathieu, 2012	Authors’ adaptation of AMSA scorecard	11	Yes		Yes		Yes	Yes		Yes	Yes	Yes		Yes	Yes	Yes		22%
Scheffer, 2017	Authors’ adaptation from AMSA scorecard and Shnier 2013	13	J	J	Yes	Yes	Yes		Yes	Yes	Yes			Yes	Yes		Yes	2%
Shnier, 2013	Authors’ adaptation from AMSA scorecard, Chimonas 2011, Mason 2011	12	J	J	Yes	Yes	Yes	Yes	Yes	Yes	Yes	Yes		Yes	Yes		Yes	30%
Yeh, 2014	AMSA and IMAP (2010 data)	11 (AMSA), 12 (IMAP)	Yes	Yes	Yes	Yes	Yes		Yes	Yes	Yes	Yes		Yes	Yes	Yes		44%

Abbreviations: AMSA, American Medical Student Association; IMAP, Institute of Medicine as a Profession; P&T, pharmacy and therapeutics; J, Joint item. In some studies the assessment tool combined multiple domains under the same item. The letter J refers to domains that were assessed jointly in that specific study. For example, when assessing policies on gifts, some studies also included meals under that item. ^a^Eight studies also assessed medical school curriculum/educational objectives in order to analyse whether students are trained on conflict-of-interest policies and on industry promotion. We have not reported data on this domain as we do not consider it to be part of the COI policy but it is still included in the total number of policy items assessed and contributes to the mean overall score. ^b^COI policies concerning membership of P&T committees or Purchasing/formulary committee participation. ^c^Additional items not reported in the Table. Carlat, 2014: access of medical device representatives to academic medical centres, extension of COI policies to community teaching affiliates and to all faculty, and enforcement; Grabitz, 2020: extension, enforcement; Scheffer, 2017: pharmaceutical industry funding of the medical school, industry educational support of residents for publication of scientific articles, and medical school activities to promote COI policies in affiliated teaching hospitals. Moreover they looked at procedures in place for monitoring and enforcement. Shnier, 2013: enforcement (oversight and sanctions). ^d^Calculated by the reviewers as mean summary score using assessment tool divided by maximum summary score possible using assessment tool. ^c^Carlat, 2016 reports on the assessment for two separate years: 2013 and 2014. Some of the domains analysed and their definition changed in 2014. In the table we report the domains assessed using the 2014 version of the assessment tool.

 In Supplementary file 3 (Table S2) we report the number of policies that received maximum score in each domain for each study. There was variation across studies and domains. Most institutions ranked poorly in the studies conducted in Europe and Australia.^12,13,22^ For example, in a study conducted among the 37 French Faculties of Medicine, only one was rated as having a model policy for two out of the 14 assessed policy domains (industry sponsorship of educational activities and industry funding of the medical school) and 36 Faculties did not have a model policy for any domains.^13^ In North America instead, more medical schools had model policies but still for several domains most institutions were not rated as having model policies.

####  Strength of the Content of COI Policies of Teaching Hospitals 

 One study analysed COI policies of 32 teaching hospitals in France^26^ using an assessment tool adapted from AMSA and other previous studies.^8,13^ Fifteen hospitals had no COI policies available on their websites and did not reply to the authors’ request for information. For 17 hospitals, policies were present for some items only (Supplementary file 4, Table S1). The mean standardised summary score was 6%; when considering only the 17 hospitals with available policies in sensitivity analysis, the score was 11%.

####  Content of COI Policies With No Assessment of Their Strength

 Eight studies examined the content of COI policies without assessing their strength. Key findings of these studies are reported in Supplementary file 4, Table S2.

 In three studies the assessment of whether the policies covered specific domains was done by the study authors based on copies of COI policies.^11,29,33^ Two of them assessed whether the policies covered specific domains using checklists designed by the authors; the checklists contained 16 and 61 items.^11,33^ For example, Lexchin assessed the content of policies that dealt with COI of investigators in Canada. All included teaching hospitals, Faculties of Medicine and their universities had policies, but they varied widely with regard to the domains addressed. Important areas were not addressed; for example, 61% of the institutions had no policies on investigators’ publication rights.^11^

 In four studies the content of the policies was based on survey responses by Deans of medical schools.^10,25,28,30^ One investigated COI policies at German medical schools, while the other three were conducted in the United States. For example, Chimonas asked Deans whether their schools had policies on 11 key items. Most of the institutions (from 55% to 70%) had policies concerning consulting, honoraria, gifts, meals, vendor access, and continuing medical education. Other policy areas were less frequently addressed; for example, only 23% of the institutions had a policy on ghostwriting.

 Finally, one study used both methods, namely survey of University officials and authors’ assessment of policies identified via institutional websites.^21^ Some discrepancies were found between the two assessment methods. For example, in the survey, most institutions (90%) reported having a policy for consulting, continuing medical education and industry-funded speaking bureaus. Instead, according to the policies identified on institutional websites, these domains were only covered by 75%, 65%, and 75% of the institutions, respectively.

####  Content of COI Policies of IRB 

 Two studies analysed the content of IRB COI policies of medical schools and hospitals.^27,35^ Detailed results are reported in Supplementary file 4, Table S3. The studies found that there is still a lack of clear guidance on how to disclose and manage COI. For example, Wolf and Zandecki reported that while 75% of the policies prohibited members from discussing or voting on a research protocol in which they had a COI, only 14% prohibited them from being reviewers of the protocol.^27^

###  Impact of COI Policies 

 None of the included studies assessed the impact of COI policies on research outputs or educational quality or content.

###  Exploration of Heterogeneity

 We found large heterogeneity of prevalence estimates with I^2^ of 98% (Figure 3). We explored reasons for heterogeneity in relation to setting, timing, and methodological quality for prevalence of general COI polices for medical schools. We did not explore heterogeneity for other types of COI policies due to the limited number of included studies for those groups. We found no indication that timing or methodological quality could explain differences in prevalence estimates (Supplementary file 5, Figures S2 and S3). However, when we stratified our analysis according to study setting, heterogeneity decreased from I^2^ of 98% in overall analysis to I^2^ of 69% for studies conducted in North America and 95% for studies conducted outside North America. When we limited the latter group to the three studies conducted in Europe, by excluding the study by Mason conducted in Australia, I^2^ decreased to 0%. Similarly, COI policies from North American institutions scored better on strength of content than policies from European institutions. As Table 2 shows, the European studies both had a summary score of 2% while in the North American studies the score ranged from 22% to 73%.

## Discussion

###  Key Findings

 Of the 22 included studies, 20 reported prevalence of COI policies (from 5% to 100%). Twenty studies assessed the content of COI policies and found that they varied widely and still had substantial shortcomings. The majority of North American institutions had COI polices in contrast to European institutions where the majority did not have COI policies. Similarly, for institutions with policies, North American policies scored better on strength of content than European institutions. None of the included studies assessed the impact of COI policies on research outputs or educational quality or content and none of the included studies investigated institutional policies in Africa, Asia or South America.

 We found a huge variability in prevalence of COI policies across countries. It is possible that some institutions that lack formal policies, still address COI on a case by case basis, and conversely the presence of a formal policy does not necessarily mean that its provisions are appropriately implemented. Enforcement mechanisms, including active oversight to ensure compliance and sanctions in case of violations are vital to ensure that policies reach their intended objectives. It is also worth mentioning that in some countries there could be laws that regulate or prohibit certain interactions with industry and these were not captured by the analyses of institutional policies. For example, in France there is a “Sunshine Policy” that requires disclosure of payments to health professionals in a publicly available database.^37^ Similarly, the analysis of institutional policies does not comprehensively capture the environment in which faculty and students operate and the difference between North American and European institutions may therefore not necessarily imply that COI have greater influence in European institutions. However, the variability we detected means that academic researchers and students are likely facing very different institutional environments when dealing with industry. Interactions with industry pose important ethical challenges and the presence of explicit institutional policies could provide a roadmap for academic researchers and students and may help to eliminate potential grey areas.

 Our findings that policies in US tended to be stricter compared to Europe could in part be explained by the AMSA scorecard. In 2007 AMSA started publishing an annual scorecard ranking US medical schools on their COI policies and the considerable media attention generated by the scorecard influenced the development of policies in several US Schools.^36^ The AMSA scorecard has also had an international impact prompting similar analyses in other countries.^8,12,13,22,23^ Similarly to the AMSA experience, the publication of the French scorecard in 2017 prompted important changes in the country with the publication of a Code of Ethics by the National Conference of Deans of Medicine and Odontology Schools^38^ that was subsequently adopted by several medical schools across the country.^39^

###  Strengths and Limitations

 To our knowledge, this is the first systematic review, to examine the prevalence, content, and impact of COI policies of medical schools and teaching hospitals. We registered our protocol before conducting the review and undertook a comprehensive search strategy. However, our review also has limitations. First, most of the studies were conducted in North America and this limits the generalisability of our findings. Second, the included studies varied in many aspects (eg, types of institutions, study period, assessment tools used) making comparison across studies challenging. While our analyses to some degree indicate that prevalence estimates are similar within geographical regions, our results are limited by few studies conducted outside North America. Third, for the North American studies, there could have been overlap between samples; however, since in many cases individual institutions and scores could not be identified, it was not possible to investigate whether differences in estimates could be due to different assessments by study authors. Fourth, the majority of studies were assessed as being of high quality, but there are no available methodological quality assessment tools specifically tailored for these types of studies and this may have affected our ability to measure the quality of included studies. Fifth, it is important to acknowledge that there are many other studies that focus on IRBs and their practices in the medical literature,^40,41^ but we only identified three that specifically dealt with their COI policies at medical schools and teaching hospitals. Sixth, while our search strategy was comprehensive we excluded 17 studies not reporting data separately for medical schools or teaching hospitals. It is possible that the study authors might have collected relevant data, but we did not contact them for additional data as this would likely require extensive recoding and analyses which was beyond the scope of our study.

###  Implications for Research

 None of the included studies assessed the impact of COI policies on research outputs or educational quality or content. The introduction of institutional COI policies on pharmaceutical promotion has been associated with reduced prescribing of newly approved psychotropic drugs^42^ and of heavily marketed and brand reformulated antidepressants.^43^ Similarly, future studies could investigate the impact of COI policies on institutional research and educational activities.

 The majority of included studies were from the United States, five from Canada, four from Europe, and one from Australia. This exposes an important knowledge gap as little research has been conducted outside of North America and no data is available from middle- or low-income settings.

 Finally, despite the great impact that the AMSA scorecard had on this field of research, it is worth mentioning also some of its limitations, which could have implications for future research. The AMSA scorecard went through a rigorous process of revisions between 2012 and 2014 thanks to the work of two committees.^36^ However, with regard to its content, the focus of the tool is mainly on interactions with industry at the individual level without considering COI at the institutional level^23^ and apart from ghostwriting the tool does not address COI and research, for example industry sponsors’ influence on study design, analysis and reporting.^44^ Moreover, the adaptation of the AMSA tool to the different local contexts – although in some cases necessary – introduced variations in the domains addressed or in their definition which made it difficult to compare the results of these studies in our review. In light of these methodological considerations, there could be a need for a new tool to assess institutional COI policies. The tool could be developed starting from the current AMSA scorecard using methodological approaches ensuring high validity and reliability.^45^

## Conclusion

 Of 22 cross-sectional studies, 20 showed that prevalence of COI policies at medical schools and teaching hospitals varied greatly in high-income countries. We found no studies estimating the prevalence of COI policies in low or middle-income countries. The content of COI policies varied widely and while most European institutions scored poorly, US medical schools have taken actions in adopting and strengthening COI policies, although many still have room for improvement. None of the included studies assessed the impact of COI policies on research outputs or educational quality or content. Interactions with industry pose important ethical challenges and the presence of explicit institutional policies could provide a roadmap for researchers and students.

## Acknowledgements

 We thank Lasse Østengaard (University of Southern Denmark) for helping us to develop the search strategy.

## Ethical issues

 Not applicable.

## Competing interests

 Authors declare that they have no competing interests.

## Authors’ contributions

 All authors contributed to study idea and design. AF conducted the literature search and screened abstracts. AF and KRH screened full texts, acquired the data, and assessed the methodological quality of included studies. AF and AL analysed the data. AF wrote the first draft of the manuscript. All authors edited drafts of this article and approved the final version.

## Funding

 No external funding. All authors were financed through institutional salaries.

## Supplementary files


Supplementary file 1. Search Strategy.

Supplementary file 2. Methodological Quality Assessment Tool Adapted From the Joanna Briggs Institute Checklist for Studies Reporting Prevalence Data.

Supplementary file 3 contains Tables S1 and S2.

Supplementary file 4 contains Tables S1-S3.

Supplementary file 5 contains Figures S1-S3.
