# Supplementary material for: Conflict of Interest Policies at Medical Schools and Teaching Hospitals: A Systematic Review of Cross-sectional Studies
Source: Int J Health Policy Manag. 2021 Mar 3;11(8):1274–85. doi: 10.34172/ijhpm.2021.12 (PMC9808354; doi:10.34172/ijhpm.2021.12)
Supplement: Supplementary file 1 — Search Strategy. [file ijhpm-11-1274-s001.pdf]

Article title: Conflict of Interest Policies at Medical Schools and Teaching Hospitals:A Systematic Review of Cross-Sectional Studies

Journal name: International Journal of Health Policy and Management (IJHPM)

Authors' information: Alice Fabbri, Kristine Rasmussen Hone, Asbjørn Hróbjartsson, Andreas Lundh

Alice Fabbri, Centre for Evidence-Based Medicine Odense (CEBMO), University of Southern Denmark and Odense University Hospital, Odense, Denmark

Kristine Rasmussen Hone, Centre for Evidence-Based Medicine Odense (CEBMO) and Cochrane Denmark, Department of Clinical Research, University of Southern Denmark, Odense, Denmark

Asbjørn Hróbjartsson, Centre for Evidence-Based Medicine Odense (CEBMO) and Cochrane Denmark, Department of Clinical Research, University of Southern Denmark, Odense, Denmark

Andreas Lundh, Centre for Evidence-Based Medicine Odense (CEBMO) and Cochrane Denmark, Department of Clinical Research, University of Southern Denmark, Odense, Denmark

Supplementary File 1. Search strategy

Search strategy for MEDLINE (adapted to search the other databases)

1. exp “Conflict of interest”
2. Conflict* of interest*. mp
3. Competing interest*.mp
4. 1 OR 2 OR 3
5. exp Financial support
6. (funding OR funder* OR sponsor* OR financ*).mp
7. 5 OR 6
8. Industr*.mp
9. Corporation*.mp
10. 8 OR 9
11. 7 AND 11
12. 4 OR 11
13. (polic* OR regulat* OR manage*)
14. (Universit* OR (teaching AND hospital*) OR ((medical OR medicine OR health) adj3 (school* OR college* OR center* OR centre* OR institution*)))
15. 12 AND 13 AND 14
