# Supplementary material for: Conflict of Interest Policies at Medical Schools and Teaching Hospitals: A Systematic Review of Cross-sectional Studies
Source: Int J Health Policy Manag. 2021 Mar 3;11(8):1274–85. doi: 10.34172/ijhpm.2021.12 (PMC9808354; doi:10.34172/ijhpm.2021.12)
Supplement: Supplementary file 2 — Methodological Quality Assessment Tool Adapted From the Joanna Briggs Institute Checklist for Studies Reporting Prevalence Data. [file ijhpm-11-1274-s002.pdf]

Article title: Conflict of Interest Policies at Medical Schools and Teaching Hospitals:A Systematic Review of Cross-Sectional Studies

Journal name: International Journal of Health Policy and Management (IJHPM)

Authors' information: Alice Fabbri, Kristine Rasmussen Hone, Asbjørn Hrobjartsson, Andreas Lundh

Alice Fabbri, Centre for Evidence-Based Medicine Odense (CEBMO), University of Southern Denmark and Odense University Hospital, Odense, Denmark

Kristine Rasmussen Hone, Centre for Evidence-Based Medicine Odense (CEBMO) and Cochrane Denmark, Department of Clinical Research, University of Southern Denmark, Odense, Denmark

Asbjørn Hrobjartsson, Centre for Evidence-Based Medicine Odense (CEBMO) and Cochrane Denmark, Department of Clinical Research, University of Southern Denmark, Odense, Denmark

Andreas Lundh, Centre for Evidence-Based Medicine Odense (CEBMO) and Cochrane Denmark, Department of Clinical Research, University of Southern Denmark, Odense, Denmark

Supplementary File 2. Methodological quality assessment tool adapted from the Joanna Briggs Institute Checklist for Studies Reporting Prevalence Data

Possible answers: High quality, low quality, unclear, not applicable

Questions	Guidance*
**1. Was the sample frame appropriate to address the target population?**	We will look at the population from which the sample is collected.<br>We will grade this as high quality if there is a clearly defined population of academic institutions from which the sample is collected.
**2. Were study participants recruited in an appropriate way?**<br>**(Selection bias:** the included participants should reflect an appropriate sample from the source population**)**	We will grade this as high quality if:<br>a) they recruited all the medical schools/teaching hospitals in the country/region;<br>b) they used a random probabilistic sample; or<br>c) they used a systematic method judged by the coder as likely to capture a broadly representative sample
**3. Were valid methods used for the identification of the outcome?**	How have the authors defined the outcomes? We will consider the outcomes that were listed in our protocol and assess how the authors have defined them in the study.<br>We will also look at the information sources/methods used for identification of outcomes.<br>In studies that aimed to identify conflict of interest policies to conduct an analysis of their content, we will assess the study as high quality if the authors used multiple methods (i.e. data sources) to identify the policies (e.g: search of website and contact with Deans). If the study only used one method, we will grade it as low quality. We will not apply this rule to surveys.
**4. Was the outcome measured reliably?**	Were the outcomes measured in a valid and reliable way? Was the outcome measured in the same way for all included institutions? Were those involved in collecting data trained in the use of the instrument/s? For example, if there was duplicate independent coding of the content of the policies, we will say ‘High quality’.

**5. Were there no missing data, and if yes, were missing data managed appropriately?**	**For surveys**, we will apply the following rules:<br><br>A low response rate for survey studies can diminish the study’s internal and external validity. The authors should clearly discuss the response rate and any reasons for non-response and compare persons/institutions in the study to those not in the study.<br><br>If the authors have included the whole population or if the response rate is >50%, we will say “High quality”.<br><br>If the response rate is <50%, the authors need to describe the differences between the respondents and the non respondents. If there seem to be no significant differences, we will say “High quality”. Instead if there are differences or if the authors have done nothing to look at whether the respondents are different from the non respondents, we will say “Low quality”.<br><br>If the response rate is not reported, we will say “Unclear”.<br><br>**For non-survey studies**, we will apply the following rules:<br><br>Studies where the authors were able to retrieve policies from all institutions in sample (for example, by searching the institutional websites of all the target insitutons) will be considered "High quality".<br><br>Studies where the authors were unable to retrieve policies from all institutions in the sample (for example, only asked the institutions to provide copies of the policies with no additional search methods and not all institutions replied) will be considered "Low quality" if <50% of the institutions in the sample provided data/responded.

*In order to develop this guidance, we also draw inspiration from BMJ 2020;368:l6925.
