# Supplementary material for: Conflict of Interest Policies at Medical Schools and Teaching Hospitals: A Systematic Review of Cross-sectional Studies
Source: Int J Health Policy Manag. 2021 Mar 3;11(8):1274–85. doi: 10.34172/ijhpm.2021.12 (PMC9808354; doi:10.34172/ijhpm.2021.12)
Supplement: Supplementary file 3 — contains Tables S1 and S2. [file ijhpm-11-1274-s003.pdf]

Article title: Conflict of Interest Policies at Medical Schools and Teaching Hospitals:A Systematic Review of Cross-Sectional Studies

Journal name: International Journal of Health Policy and Management (IJHPM)

Authors' information: Alice Fabbri, Kristine Rasmussen Hone, Asbjørn Hróbjartsson, Andreas Lundh

Alice Fabbri, Centre for Evidence-Based Medicine Odense (CEBMO), University of Southern Denmark and Odense University Hospital, Odense, Denmark

Kristine Rasmussen Hone, Centre for Evidence-Based Medicine Odense (CEBMO) and Cochrane Denmark, Department of Clinical Research, University of Southern Denmark, Odense, Denmark

Asbjørn Hróbjartsson, Centre for Evidence-Based Medicine Odense (CEBMO) and Cochrane Denmark, Department of Clinical Research, University of Southern Denmark, Odense, Denmark

Andreas Lundh, Centre for Evidence-Based Medicine Odense (CEBMO) and Cochrane Denmark, Department of Clinical Research, University of Southern Denmark, Odense, Denmark

Supplementary File 3

Table S1. Mean standardised summary score calculated by the reviewers

**Study**	**All institutions included**	**Sensitivity analysis** (excluding institutions without COI policies)
**Carlat, 2016**	2013: 73%<br>2014: 56%	Number of institutions without COI policies not reported.
**Chimonas, 2011**	40%	Number of institutions without COI policies not reported.
**Chimonas, 2013**	2008: 27%<br>2011: 56%	95% of the institutions have a policy in 2011<br>Average score for 2011: 59%
**Grabitz, 2020**	2%	31%
**Mason, 2011**	26%	31%
**Mathieu, 2012**	22%	22% (all schools had policies)
**Scheffer, 2017**	2%	7%
**Shnier, 2013**	30%	32%
**Yeh, 2014**	AMSA: 44%	44% (all included institutions had policies as confirmed by the author)

Table S2. Number of 'model' policies for each policy domain for the nine studies that used assessment tools to assess the strength of the policies (a)

Study ID / Policy items	Carlat, 2016 (b)	Chimonas, 2011 (c)	Chimonas, 2013 (c)	Grabitz, 2020 (d)	Mason, 2011	Mathieu, 2012	Scheffer, 2017 (e)	Shnier, 2013	Yeh, 2014 (f)
**Gifts**	2013: 93/158 (59%) (Joint)<br><br>2014 (does not include meals): 79/160 (49%)	23/77 (30%)	2008: 10/77 (13%)<br>2011: 44/127 (35%)	1/38 (3%)	0/20 (0%) (Joint)	12/16 (75%)	0/37 (0%) (Joint)	4/17 (24%) (Joint)	AMSA: 3 (1.5, 3)<br>IMAP: 2 (2, 3)
**Meals**	2013: 93/158 (59%) (Joint)<br><br>2014: 24/160 (15%)	20/77 (26%)	2008: 16/77 (21%)<br>2011: 62/127 (49%)	0/38 (0%)	0/20 (0%) (Joint)		0/37 (0%) (Joint)	4/17 (24%) (Joint)	AMSA: n/a<br>IMAP: 2 (2,3)
**Sales reps**	2013: 4/158 (3%)<br>2014: 9/160 (6%)	15/77 (19%)	2008: 8/77 (10%)<br>2011: 21/127 (17%)	0/38 (0%)		0/16 (0%)	0/37 (0%)	0/17 (0%)	AMSA: 1.5 (1.5, 1.5)<br>IMAP: 2 (2, 2)
**Honoraria**		2/77 (3%)	2008: 1/77 (1%)<br>2011: 8/127 (6%)	1/38 (3%) (Joint)			0/37 (0%)	2/17 (12%)	AMSA: n/a<br>IMAP: 1 (0,2)
**Consulting**	2013: 71/158 (45%)<br>2014: 26/160 (16%)	1/77 (1%)	2008:0/77 (0%)<br>2011: 4/127 (3%)	0/38 (0%)	0/20 (0%)	11/16 (69%)	0/37 (0%)	3/17 (18%)	AMSA: 1.5 (1.5,3)<br>IMAP: 1 (1, 2)
**Industry scholarships**	2013: 121/158 (77%)<br>2014: 3/160 (2%) (Joint)	14/77 (18%) (Joint)	2008: 5/77 (6%)	1/38 (3%) (Joint)		0/16 (0%)		6/17 (35%)	AMSA: 1.5 (0, 1.5)<br>IMAP: 3 (3,3)

			2011:26/127 (20%)						
**Ghostwriting**	2013: N/A, 2014: 105/160 (66%)	17/77 (22%)	2008: 16/77 (21%) 2011:81/127 (64%)	0/38 (0%)			0/37 (0%)	8/17 (47%)	AMSA: n/a IMAP: 0 (0, 3)
**Speakers' bureaus**	2013: 43/158 (27%), 2014: 79/160 (49%)	3/77 (4%)	2008: 4/77 (5%) 2011:30/127 (24%)	0/38 (0%)	0/20 (0%)	4/16 (25%)	0/37 (0%)	2/17 (12%)	AMSA: 1.5 (0, 1.5) IMAP: 1 (1,2)
**Disclosure**	2013: 39/158 (25%) 2014: 51/160 (32%)			1/38 (3%)	0/20 (0%)	0/16 (0%)	0/37 (0%)	1/17 (6%)	AMSA: 1.5 (0, 1.5) IMAP: n/a
**Samples**		13/77 (17%)	2008: 7/77 (9%) 2011:35/127 (28%)			0/16 (0%)		0/17 (0%)	AMSA: 1,5 (0, 1.5) IMAP: 1 (1, 3)
**Attendance of promotional events**	2013: N/A 2014: 25/160 (16%)			0/38 (0%)					
**Industry sponsorship of educational events**	2013: 101/158 (64%) 2014: 5/160 (3%)	12/77 (16%)	2008: 6/77 (8%) 2011:16/127 (13%)	0/38 (0%)	0/20 (0%)	0/16 (0%)	1/37 (3%)	3/17 (18%)	Industry sponsorship of continuing medical education events: AMSA: n/a IMAP: 1 (1, 1) On-site educational events:

									AMSA 1.5 (1.5, 1.5) IMAP: n/a
**Travel / Off-site education**	2013: 121/158 (77%) 2014: 3/160 (2%) (Joint)	14/77 (18%) (Joint)	2008: 6 /77 (8%) 2011: 34/127 (27%)		0/20 (0%)	0/16 (0%)	0/37 (0%)	3/17 (18%)	AMSA: 3 (1.5, 3) IMAP: 2 (1, 3)
**Pharmacy and Therapeutic Committees/Purchasing**		17/77 (22%)	2008: 9/77 (12%)<br>2011: 43/127 (34%)			0/16 (0%)			AMSA: 3 (1.5, 3) IMAP: 0 (0, 2)
**Other**	Medical device reps: n/a in 2013, 91/160 (57%) in 2014<br><br>Extension of COI policies: N/A in 2013, 50/160 (31%) in 2014<br><br>Enforcement: N/A in 2013, 126/160 (79%) in 2014			Extension of policy: 1/38 (3%)<br><br>Enforcement: 1/38 (3%)			Pharmaceutical industry funding of the medical school: 1/37 (3%)<br><br>Industry educational support of residents for publication of scientific articles. 0/37 (0%)<br><br>Medical school activities to promote COI policies in affiliated teaching hospitals 0/37 (0%)<br><br>Oversight: 0/37 (0%) (g)<br><br>Sanctions: 0/37 (0%) (g)	Oversight: 15/17 (g)<br><br>Sanctions: 10/17(g)	

a) By "model policy" we mean the policies that received the highest score according to the assessment tool used in each study. Some studies call them 'stringent' or 'restrictive' policies instead of 'model' policies. As in Table 2, we do not report on the item related to COI curriculum (which was measured in several studies) as it is not relevant to our review questions.
b) Carlat, 2016 conducted an analysis of the COI policies by medical school type and geographic region. Allopathic schools were more likely to have complete COI policies compared to osteopathic schools. No differences were observed between geographic regions.

c) Chimonas, 2011 and Chimonas, 2013 analysed whether the strength of the policies changed by school status (public or private), hospital ownership/affiliation, and level of NIH funding. No differences were observed for the first two factors; instead greater NIH funding was associated with more stringent policies.
d) Grabitz, 2019: the authors identified (and assessed the content of) COI policies only for 2/38 German medical schools; the other 36 schools scored 0 on all the items as no policies were identified.
e) Scheffer, 2017: the authors found that 2/37 French medical schools had COI policies and 9/37 had either a COI policy or had introduced related curriculum; the other 28 medical schools scored 0 on all the items.
f) Yeh, 2014 does not report the number of policies with the highest score. It reports instead median (IQR) for two studies previously conducted by AMSA (American Medical Student Association) and IMAP (Institute of Medicine as a Profession).
g) In this case the answer was Yes/No and not a score. Since 'Yes' would be the answer for a good policy, we report the proportion of policies who received a "Yes" for this item.

Joint: As explained in Table 2 (main manuscript), in some studies the assessment tool combined multiple items under the same domain. For example, when assessing policies on gifts, several studies included also meals. In those cases, we report the data twice (under each of the relevant domain) and we add the word "joint" to flag this.
