# Supplementary material for: Conflict of Interest Policies at Medical Schools and Teaching Hospitals: A Systematic Review of Cross-sectional Studies
Source: Int J Health Policy Manag. 2021 Mar 3;11(8):1274–85. doi: 10.34172/ijhpm.2021.12 (PMC9808354; doi:10.34172/ijhpm.2021.12)
Supplement: Supplementary file 4 — contains Tables S1-S3. [file ijhpm-11-1274-s004.pdf]

Article title: Conflict of Interest Policies at Medical Schools and Teaching Hospitals:A Systematic Review of Cross-Sectional Studies

Journal name: International Journal of Health Policy and Management (IJHPM)

Authors' information: Alice Fabbri, Kristine Rasmussen Hone, Asbjørn Hrobjartsson, Andreas Lundh

Alice Fabbri, Centre for Evidence-Based Medicine Odense (CEBMO), University of Southern Denmark and Odense University Hospital, Odense, Denmark

Kristine Rasmussen Hone, Centre for Evidence-Based Medicine Odense (CEBMO) and Cochrane Denmark, Department of Clinical Research, University of Southern Denmark, Odense, Denmark

Asbjørn Hrobjartsson, Centre for Evidence-Based Medicine Odense (CEBMO) and Cochrane Denmark, Department of Clinical Research, University of Southern Denmark, Odense, Denmark

Andreas Lundh, Centre for Evidence-Based Medicine Odense (CEBMO) and Cochrane Denmark, Department of Clinical Research, University of Southern Denmark, Odense, Denmark

Supplementary File 4.

Table S1. Overview of COI policy scores for 17 French teaching hospital (Guy-Coichard)

**Policy Domain**	**Results***
Management of gifts and benefits	Score of 1: 4/17, Score of 2: 8/17, Score of 3: 1/17
Access of representatives of pharmaceutical companies	Score of 1: 3/17, Score of 2: 2/17
Access of representatives of medical equipment, biology and imaging companies	Score of 1: 3/17, Score of 2: 2/17
Advisory or speaking activities	Score of 1: 4/17, Score of 2: 8/17
Promotional presentation or speeches	Score of 1: 3/17
Promotional events funded by companies	Score of 3: 1/17
Medical conferences funded by companies	Score of 1: 1/17
Research funding	Score of 1: 1/17, Score of 3: 1/17
Hospital service associations	Score of 2: 2/17
Frameworks of market surveys	Score of 3: 2/17
Procurement of medicines or medical devices	Score of 1: 1/17, Score of 2: 4/17, Score of 3: 1/17
COI education for teaching hospital staff	Score of 1: 1/17 Score of 2: 1/17
Extension of rules to all actors in hospital	Score of 1: 3/17 Score of 2: 1/17
Governance rules	Score of 2: 3/17
Monitoring the application of rules and sanctions	Score of 1: 2/17, Score of 2: 1/17
Authorities responsible for monitoring	Score of 1: 1/17, Score of 3: 2/17

*Scoring system used by the authors: *"0: the hospital has no policy for this criterion, or it is not accessible; 1: The hospital has an explicit policy, communicated to staff, but no additional information is available on regulatory and legal obligations for this criterion; 2: the hospital policy is limited in scope, or monitoring is insufficient; 3: the hospital has an explicit policy on this issue at a high standard, reflecting standards in French law and contractual agreements between health authorities and health industry associations".* The items and the hospitals that do not appear in the Table have a zero score.

Table S2. Key findings of articles that assessed content of COI policies via coding of the documents by the authors or surveys of University officials.

Study	Methods	Key results
**CODING OF THE POLICIES DONE BY THE AUTHORS**		
Lexchin, 2008	The authors used an instrument containing 61 items related to financial COI. The coding instrument contains six broad domains:<br>1. Definitions<br>2. Interests requiring disclosure<br>3. Who reviews and makes Decisions about financial COI<br>4. Strategies for managing financial COI<br>5. Disclosure details<br>6. Publication rights.<br>The authors abstracted data from the policies using the above mentioned instrument. No scoring of the strength of the policy.	Domain 1 Definitions<br>Policies applicable to: staff 63/74 (85%), trainees/students 56/74 (76%), post-docs 58/74 (78%), academic staff/faculty 74/74 (100%), other 27/74 (36%)<br>Definitions of COI provided 58/74 (78%)<br>Examples of COI provided 46/74 (62%)<br>Definition of financial interest provided 27/74 (36%)<br>Types of COI regulated by the policy: financial COI related to personal financial interests 70/74 (95%), Financial COI related to family's financial interests 61/74 (82%), Conflicts of commitment (conflicts re: time, outside activities) 58/74 (78%), financial COI related to hiring of person's family 36/74 (49%), financial COI related to trainees/education 32/74 (43%)<br><br>Domain 2: Interests requiring disclosure<br>Financial compensation: salary 32/74 (43%), honoraria/donations 36/74 (49%), consulting fees 40/74 (54%)<br>Research sponsorship: grant 11/74 (15%), Research support/overhead 18/74 (24%), contracts 42/74 (57%), per capita payments for recruiting research participants 17/74 (23%)<br>Indirect financial benefits: intellectual property right 42/74 (57%), stock or stock options 41/74 (55%), gifts 47/74 (64%)<br>Other benefits: management position in company 31/74 (42%), advisory board memberships in company 32/74 (43%), establishing a corporation/spin-off company and related activities 9/74 (12%), speakers list of a company 10/74 (14%)<br><br>Domain 3: Who reviews and makes Decisions about financial COI<br>Disclosed to: Department Chair/supervisor 52/74 (70%), VP/Chief research or VP academic 35/74 /47%), Dean of faculty 27/74 (36%), Legal counsel 6/74 (8%), REB 31/74 (42%), Committee 16/74 (22%), Other 40/74 (54%),<br>Decision maker: Department Chair/supervisor 41/74 (55%), VP/Chief research or VP academic 30/74 (41%), Dean of faculty 27/74 (36%), Legal counsel 6/74 (8%), REB 24/74 (32%), Committee 18/74 (24%), Other 38/74 (51%)<br><br>Domain 4: Strategies for managing FCOI<br>Disclosure of information to the centre 71/74 (96%)<br>Disclosure to public 21/74 (28%)<br>Disclosure to funder 16/74 (22%)<br>Monitoring/oversight 28/74 (38%)<br>Disqualification from/discontinuation of research 29/74 (39%)<br>Divestiture/ prohibition of financial interests 19/74 (26%)<br>Disclosure to research participants 25/74 (34%)

		Other 32/74 (43%)<br><br><u>Domain 5: Disclosure details</u><br>Disclosure is independent in nature 51/74 (69%)<br>Disclosure must occur prior to the commencement of the activity 47/74 (64%)<br>Regular review of potential conflicts of interest is required 41/74 (55%)<br>Committee on conflicts of interest exists at the institution: 23/74 (31%)<br>Sanctions described for those who fail to comply 39/74 (53%)<br>Appeals process described 32/74 (43%)<br>Rebuttable presumption that financially interested individuals may not conduct research without undergoing review and receiving permission 16/74 (22%)<br>FCOI review must be done prior to REB approval 25/74 (34%)<br>REB members must disclose any potential FCOI they may have with research under review 21/74 (28%)<br><br><u>Domain 6: publication rights</u><br>Publication rights addressed 29/74 (39%)<br>Specified time limit related to publication 9/74 (12%)<br>Specified exceptions related to publications 11/74 (15%)
Lo, 2000	The authors assessed the policies of 10 medical schools using legal principles for the interpretation of contracts and statutes. They specifically looked at prohibited interests and which interest need to be disclosed.	<u>Interests that must be disclosed</u><br>1. Stock 10/10<br>2. Stock option 10/10<br>3. Income 10/10<br>4. Loan or gift 8/10<br>5. Decision-making position 7/10<br>Note: the authors describe the cut-offs for disclosure. (data not reported here)<br><br><u>Person with interest requiring disclosure:</u><br>1. Faculty member: 10/10<br>2. Immediate family 10/10<br>3. Selected research staff: 3/10<br>4. All research staff : 4/4<br>5. Trainees: 4/4<br><br><u>Party to which disclosure must be made</u>:<br>1. University official or committee 10/10<br>2. Institutional review board 6/10<br>3. Research subjects 2/10

		4. Professional community (in publications or presentations): 4/10<br><br><u>Penalties for Noncompliance</u> 7/10<br>The sanctions included *"censure, suspension of grants and of IRB approval of studies, nonrenewal of appointment, and dismissal. The application of these penalties is discretionary"*.<br><br><u>Prohibited interest:</u><br>1. Stock: 4/10<br>2. Stock options: 4/10<br>3. Consulting fee : 3/10<br>4. Decision-making position: 1/10
Rochon, 2010	The authors conducted a content analysis of 38 policies of medical schools, teaching hospitals and their parent universities using a coding instrument containing 16 items relevant to institutional financial COI	<u>Definitions (14/38, 37%)</u><br>1. Policy title includes "institutional COI" 6/38 (16%)<br>2. Definition of institutional COI 4/38 (11%)<br>3. Definition of financial COI 8/38 (21%)<br><br><u>Categories of institutional conflicts covered (36/38, 95%)</u><br>1. Institution 7/38 (18%)<br>2. Senior institutional officials 35/38 (92%)<br><br><u>Scope of financial interests covered (22/38, 58%)</u><br>1. Royalties from sale of the investigational product that is the subject of research 8/38 (21%)<br>2. Equity interest or an entitlement to equity of any value in a non-publicly traded sponsor of human subjects research at the institution 6/38 (16%)<br>3. Ownership interest or an entitlement to equity in a publicly traded sponsor of human subjects research at the institution 6/38 (16%)<br>4. Institutional officials with direct responsibility for human subjects research hold a significant financial interest in a commercial research sponsor or investigational product 21/38 (55%)<br><br><u>Management of potential institutional financial COI (12/38, 32%)</u><br>1. Institutional COI committee exists: 4/38 (11%)<br>2. Disclosure/reporting of institutional COI required: 7/38 (18%)<br>3. Disclosure to the REB required: 2/38 (5%)<br>4. Rebuttable presumption against conduct of human subjects research when institutional level financial COI exists: 1/38 (3%)<br>5. Procedure for conducting institutional-level audits for COI: 3/38 (8%)<br>6. Technology transfer at the institution separate from the human subjects research administration: 2/38 (5%)<br>7. Endowment/investments managed externally through legally separate organizations: 0

		Overall (Number of items covered out of 16)<br>1. Mean: 3.2 (SD: 2.6)<br>2. Mean percentage: 20%<br>3. Median: 2 (range: 0-10)
**SURVEYS OF DEANS**		
Chimonas 2011	Deans and compliance officers at 125 U.S. medical schools were asked whether their institutions had formal policies in 11 areas.	Does your institution have formal policies that cover the following areas? (N=77)<br><br>1. Gifts: 44 (57%) Yes, 11 (14%) No, 22 (29%) in progress<br>2. Meals: 44 (57%) Yes, 12 (16%) No, 21 (27%) in progress<br>3. Vendor access: 42 (55%) Yes, 17 (22%) No, 18 (23%) in progress<br>4. Honoraria: 54 (70%) Yes, 6 (8%) No, 17 (22%) in progress<br>5. Industry funding for continuing medical education: 42 (55%) Yes, 14 (18%) No, 21 (27%) in progress<br>6. Consulting: 54 (70%) Yes, 6 (8%) No, 17 (22%) in progress<br>7. Scholarships/fellowships/travel: 34 (44%) Yes, 21 (27%) No, 22 (29%) in progress<br>8. Ghostwriting:18 (23%) Yes, 38 (49%) No, 21 (27%) in progress<br>9. Speakers' bureaus: 31 (40%) Yes, 24 (31%) No, 22 (29%) in progress<br>10. Samples: 34 (44%) Yes, 24 (31%) No, 19 (25%) in progress<br>11. Pharmacy and Therapeutic committees: 32 (42%) Yes, 32 (42%) No, 13 (17%) in progress
Ehringhaus, 2008	Survey of deans of 125 medical schools in the United States.<br>The survey covered four domains: whether medical schools have adopted institutional COI policies, the scope of these policies with regard to covered entities and financial interests; the existence of organizational structures as means to address ICOI; and the institutions' linkages	Policy Adoption Status and Scope<br><br>1. Has your institution adopted a conflicts policy covering the financial interests held by the institution? Yes: 30/79 (38%) Working on a policy: 29/79 (37%) Not working on a policy/Don't know: 20/79 (25%)<br><br>2. Has your institution adopted a conflicts policy covering the financial interests held by Senior institutional officials? Yes: 55/78 (71%) Working on a policy: 9/78 (12%) Not working on a policy/Don't know: 14/78 (18%)<br><br>3. Has your institution adopted a conflicts policy covering the financial interests held by midlevel institutional officials? Yes: 55/80 (69%) Working on a policy: 12/80 (15%) Not working on a policy/Don't know: 13/80 (16%)<br><br>4. Has your institution adopted a conflicts policy covering the financial interests held by institutional review board members? Yes: 62/77 (81%) Working on a policy: 6/77 (8%) Not working on a policy/Don't know: 9/77 (12%)

	between ICOI and their institutional review boards.	5. Has your institution adopted a conflicts policy covering the financial interests held by governing board members? Yes: 51/77 (66%) Working on a policy: 2/77 (3%) Not working on a policy/Don't know: 24/77 (31%)<br><br><u>Which of the following financial interests held by your institution are considered potential institutional COI?</u><br>Of the institutions that have adopted policies covering the institution's own financial interests (n=30):<br>24 (80%) respondents have adopted policies that cover royalties,<br>22 (73%) cover milestone payments,<br>27 (90%) cover equity in non-publicly held companies,<br>23 (77%) cover equity in publicly held companies,<br>22 (73%) cover receipt of substantial gifts from potential commercial sponsors of research.<br><br><u>Are the following financial interests held by an institutional official with research oversight/management responsibility considered potential institutional COI?</u><br>43/55 (78%) institutions that have adopted policies covering senior and midlevel officials considered the financial interest of an institutional research official in a commercial sponsor of research at that institution to be a potential ICOI.<br>43/55 (78%) consider that a significant financial interest held by a senior official in a product that is the subject of research an institutional COI. For the same interest held by midlevel officials, the prevalence is 42/55 (76%)<br><br>The authors also explored whether policies varied between public and private institutions and by NIH funding. No differences were observed.
Lieb, 2014	Survey of student affairs' Deans	1) Contacts between industry and medical students are prohibited: 1/30 (Aachen University Hospital)<br><br>2) Presentations by industry are permitted only after consultation with the staff of the Faculty and can be forbidden by the Dean: 1/30 (Aachen University)<br><br>The dean of the medical faculty in Dresden stated that there is a policy on conflicts of interest, no further information were provided on its content.
Weinfurt, 2010	Survey of officials at academic medical centres involved in phase 3 clinical trials	1) Are Investigators required to report financial relationships in clinical research? 61/61 (100%)<br><br>2) Review of financial relationships includes consideration of reasonableness of per capita payment amounts, 25/61 (42%)<br><br>3) Institutions has non employee investigators: 31/61 (51%) Institution reviews financial relationships of nonemployee investigators, 28/31 (90%) If no (n=2), other institution or office reviews financial relationships of nonemployee investigators, 2/2 (100%)<br><br>4) Institution uses monetary threshold below which there is no review of investigators' financial relationships: 30/61 (49%)

		Out of the sites that reported a monetary threshold (n=30), Institution uses NIH threshold: 22/30 (73%)<br><br>5) Institution prohibits investigator financial relationships (Out of sites that reported a prohibition of investigator financial relationships, n=23)<br>a)Consulting, 7/23 (30%)<br>b)Equity, 12/23 (52%)<br>c)Per capita payments 7/23 (30%)<br>d) Other 16/23 (70%)
**MIXED (Survey of University officials and authors' assessment)**		
Klein, 2018	Used 19 domains inspired by AMSA and then compared AMSA results in 2014 for 173 institutions with:<br>1) Answers on the 19 policy domains provided by the University officials of the 20 institutions included in this study<br>2) Scoring of the same domains done by the authors on the policies found on websites of the 20 institutions included in the study.	1. Gifts: Survey: N/A, Websites: 18/20 (90%)<br>2. Meals: Survey: N/A, Websites: 16/20 (80%)<br>3. CME: Survey 18/20 (90%), Websites 13/20 (65%)<br>4. Sales reps: Survey: N/A, Websites: 12-13/20 (60-65%)<br>5. Device reps: Survey: N/A, Websites: 13-14/20 (65-70%)<br>6. Consulting: Survey: 18/20 (90%) Websites: 15/20 (75%)<br>7. Scholarships: Survey: N/A Websites: 8/20 (40%)<br>8. Ghostwriting: Survey: N/A Websites: 9/20 (45%)<br>9. Speakers' bureaus: Survey: 18/20 (90%) Websites: 15/20 (75%)<br>10. Disclosure: Survey: N/A Websites: 17/20 (85%)<br>11. Sampling or distribution: Survey: 13/20 (65%) Websites: 12-13/20 (60-65%)<br>12. Promotional events: 2/20 (24%), 13/20 (65%)<br>13. Research Funding or Trials: Survey: 13/20 (65%), Websites: 19/20 (95%)<br>14. Campus based clinics or offices (medications): Survey: 13/20 (65%) , Websites:12/20 (60%)<br>15. Clinical sites for students (medications): Survey: 5/20 (25%); Websites: 11/20 (55%)<br>16. Clinical sites off campus (medications): Survey: 8/20 (40%), Websites: 8/20 (40%)<br>17. Policy extension to all clinicians: Survey: 7-18 (65-90%), Websites: 5/20 (25%)<br>18. Enforcement: Survey N/A, Websites: 12/20 (60%)<br><br>The domain on COI curriculum is not reported.

Table S3. Content of IRB COI Policies

Study	Key findings
**Campbell, 2006**	Does the IRB have a formal written definition of what constitutes a COI?<br>Yes: 45.8%<br>No: 12.1%<br>Do not know: 42.2%<br><br><u>Disclosure:</u><br>Is there a defined process for members to disclose to the IRB financial and other relationships with industry?<br>Yes: 67.0%<br>No/Did not know: 33.0%<br><br>What does the IRB do regarding the disclosure of relationships with industry?<br>49.1% required to fill out a form documenting their relationships when joining the IRB<br>45.6% relationships openly discussed by the IRB<br><br>The article contains more data on whether the respondent had COI with industry during the most recent year of IRB service, how these was managed and the effect of industry relationships on the activity of the IRB. (data not relevant for our review question)
**Wolf, 2007**	 <u>Definition of COI:</u><br>73/92 IRBs (79%) with written policies define COI<br>19/92 (21%) of IRBs with written policies provide no definition of COI<br><br><u>Financial interests</u>:<br>51/73 (70%) of IRBs that define COI include financial interests in their definition.<br>How IRBs define a financial COI with a research project:<br>a) Any financial interest: 10 IRB policies<br>b)Significant financial interest: b1: >$10,000 payments or equity: 22 policies b2: >$25,000 payments, > $50,000 equity: 1 policy b3: Undefined: 4 policies<br>c)No definition : 14 policies<br><br><u>Procedures to collect information about COI</u><br>18/90 (20%) have procedures for collecting COI information in writing<br><br><u>Extension of the policy</u><br>91 (99%) refer to IRB members<br>1 (1%) refer only to IRB Chairs.<br>13 (14%) extend to IRB staff

	18 (20%) apply explicitly to ad hoc reviewers or consultants<br>4 (4%) apply to guests (but not to staff or consultants)<br><br><u>How to deal with COI of IRB members:</u><br>1) <u>Protocol reviewer</u><br>24/92 (26%) address COI related to serving as protocol reviewers<br>13/92 (14%) prohibit members from being reviewers of protocols in which they have COI.<br><br>2) <u>Protocol discussion</u><br>69/92 (75%) require to leave the meeting during the discussion of a protocol in which they have COI.<br><br>3) <u>Voting</u><br>76/92 (83%) require to recuse from voting on a protocol in which they have COI<br><br><u>Sanction</u>:<br>4 list sanctions for failure to comply with the COI policy<br><br>The authors also compared the existence and content of IRB COI policies by amount of NIH funding, region, and institution type (public versus private). Institutions receiving the most NIH funding were more likely to: a) define COI (p value = 0.001) and b) indicate that a conflicted member may provide information to IRB (p value = 0.006). Differences by region were found with regard to whether the policies apply to consultants (p value = 0.013).
