# Supplementary material for: Conflict of Interest Policies at Medical Schools and Teaching Hospitals: A Systematic Review of Cross-sectional Studies
Source: Int J Health Policy Manag. 2021 Mar 3;11(8):1274–85. doi: 10.34172/ijhpm.2021.12 (PMC9808354; doi:10.34172/ijhpm.2021.12)
Supplement: Supplementary file 5 — contains Figures S1-S3. [file ijhpm-11-1274-s005.pdf]

Article title: Conflict of Interest Policies at Medical Schools and Teaching Hospitals:A Systematic Review of Cross-Sectional Studies

Journal name: International Journal of Health Policy and Management (IJHPM)

Authors' information: Alice Fabbri, Kristine Rasmussen Hone, Asbjørn Hróbjartsson, Andreas Lundh

Alice Fabbri, Centre for Evidence-Based Medicine Odense (CEBMO), University of Southern Denmark and Odense University Hospital, Odense, Denmark

Kristine Rasmussen Hone, Centre for Evidence-Based Medicine Odense (CEBMO) and Cochrane Denmark, Department of Clinical Research, University of Southern Denmark, Odense, Denmark

Asbjørn Hróbjartsson, Centre for Evidence-Based Medicine Odense (CEBMO) and Cochrane Denmark, Department of Clinical Research, University of Southern Denmark, Odense, Denmark

Andreas Lundh, Centre for Evidence-Based Medicine Odense (CEBMO) and Cochrane Denmark, Department of Clinical Research, University of Southern Denmark, Odense, Denmark

Supplementary File 5.

Figure S1. Forest plot of prevalence of conflicts of interest policies by setting (North America in green versus other settings in red)

Study	Policy present	Total	Proportion (95% CI)	
**General policies at medical schools**				
Yeh, 2014*	121	121	1	(0.97 to 1)
Mathieu, 2012	16	16	1	(0.79 to 1)
Chimonas, 2013	127	133	0.95	(0.90 to 0.98)
Shnier, 2013	16	17	0.94	(0.71 to 1)
Klein, 2018	18	20	0.90	(0.68 to 0.99)
Mason, 2011	17	20	0.85	(0.62 to 0.97)
Lieb, 2014**	2	30	0.07	(0.01 to 0.22)
Scheffer, 2017	2	37	0.05	(0.01 to 0.18)
Grabitz, 2020	2	38	0.05	(0.01 to 0.18)
**General policies at teaching hospitals**				
GuyCoichard, 2019	17	32	0.53	(0.35 to 0.71)
**Policies for Institutional Review Boards**				
Ehringhaus, 2008	62	77	0.81	(0.70 to 0.89)
Wolf, 2007	92	124	0.74	(0.66 to 0.82)
**Policies for investigators**				
Lexchin, 2008	74	74	1	(0.95 to 1)
Lo, 2000	10	10	1	(0.69 to 1)
Weinfurt, 2010	59	61	0.97	(0.89 to 1)
**Polices for medical students**				
Sierles, 2005	10	99	0.10	(0.05 to 0.18)

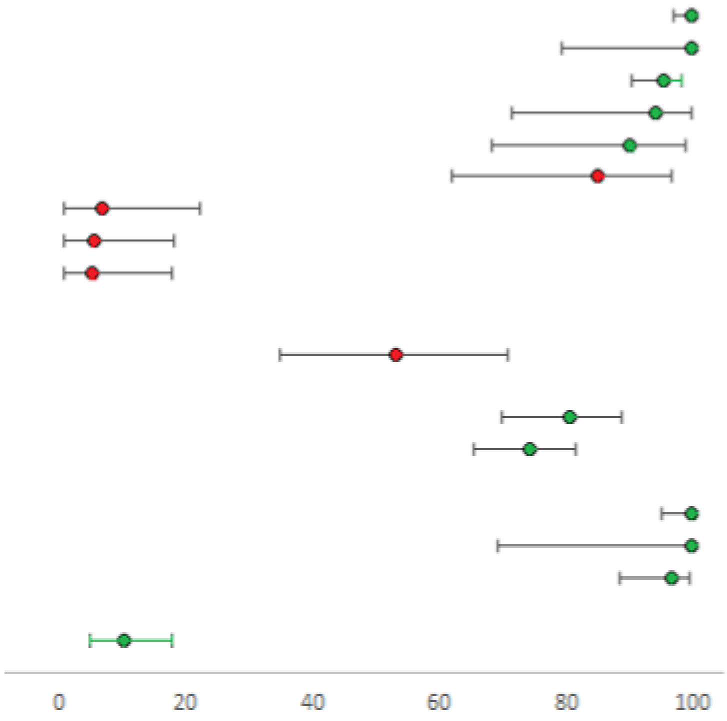

Heterogeneity: North American general medical school policies I^2: 69%. Other general medical school policies: I^2 95% (0% without Australian study by Mason et al)

*Data provided by the author.
**Lieb, 2014 asked about the existence of a general COI policy and/or a policy on interactions between medical students and industry.
I^2: Heterogeneity described by calculating I^2

Figure S2. Forest plot of prevalence of conflicts of interest policies by time of publication (before 2013 in green versus during or after 2013 in red)

Study	Policy present	Total	Proportion (95% CI)	
**General policies at medical schools**				
Yeh, 2014*	121	121	1	(0.97 to 1)
Mathieu, 2012	16	16	1	(0.79 to 1)
Chimonas, 2013	127	133	0.95	(0.90 to 0.98)
Shnier, 2013	16	17	0.94	(0.71 to 1)
Klein, 2018	18	20	0.90	(0.68 to 0.99)
Mason, 2011	17	20	0.85	(0.62 to 0.97)
Lieb, 2014**	2	30	0.07	(0.01 to 0.22)
Scheffer, 2017	2	37	0.05	(0.01 to 0.18)
Grabitz, 2020	2	38	0.05	(0.01 to 0.18)
**General policies at teaching hospitals**				
GuyCoichard, 2019	17	32	0.53	(0.35 to 0.71)
**Policies for Institutional Review Boards**				
Ehringhaus, 2008	62	77	0.81	(0.70 to 0.89)
Wolf, 2007	92	124	0.74	(0.66 to 0.82)
**Policies for investigators**				
Lexchin, 2008	74	74	1	(0.95 to 1)
Lo, 2000	10	10	1	(0.69 to 1)
Weinfurt, 2010	59	61	0.97	(0.89 to 1)
**Polices for medical students**				
Sierles, 2005	10	99	0.10	(0.05 to 0.18)

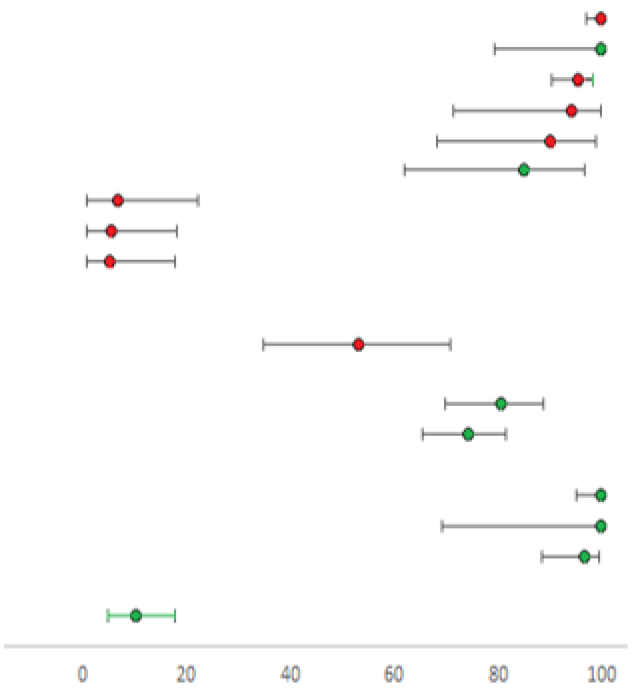

Heterogeneity: Before 2013 general medical school policies: I^2: 70%; After 2013 general medical school policies: I^2 99%

*Data provided by the author.
**Lieb, 2014 asked about the existence of a general COI policy and/or a policy on interactions between medical students and industry.
I^2: Heterogeneity described by calculating I^2

Figure S3. Forest plot of prevalence of conflicts of interest policies by study quality (high quality in green versus low quality in red)

Study	Policy present	Total	Proportion (95% CI)	
**General policies at medical schools**				
Yeh, 2014*	121	121	1	(0.97 to 1)
Mathieu, 2012	16	16	1	(0.79 to 1)
Chimonas, 2013	127	133	0.95	(0.90 to 0.98)
Shnier, 2013	16	17	0.94	(0.71 to 1)
Klein, 2018	18	20	0.90	(0.68 to 0.99)
Mason, 2011	17	20	0.85	(0.62 to 0.97)
Lieb, 2014**	2	30	0.07	(0.01 to 0.22)
Scheffer, 2017	2	37	0.05	(0.01 to 0.18)
Grabitz, 2020	2	38	0.05	(0.01 to 0.18)
**General policies at teaching hospitals**				
GuyCoichard, 2019	17	32	0.53	(0.35 to 0.71)
**Policies for Institutional Review Boards**				
Ehringhaus, 2008	62	77	0.81	(0.70 to 0.89)
Wolf, 2007	92	124	0.74	(0.66 to 0.82)
**Policies for investigators**				
Lexchin, 2008	74	74	1	(0.95 to 1)
Lo, 2000	10	10	1	(0.69 to 1)
Weinfurt, 2010	59	61	0.97	(0.89 to 1)
**Polices for medical students**				
Sierles, 2005	10	99	0.10	(0.05 to 0.18)

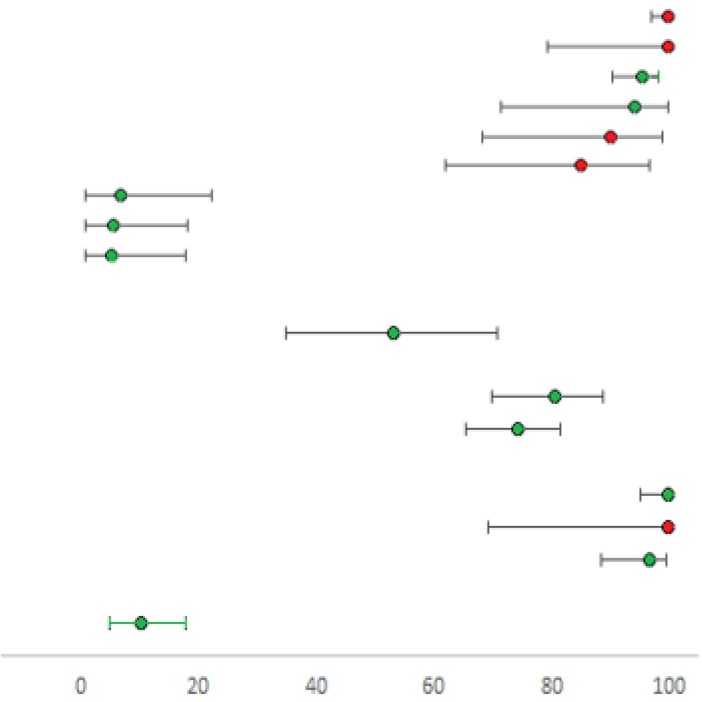

Heterogeneity: High quality general medical school policy studies: I^2: 99%; Low quality general medical school policy studies: I^2: 80%

*Data provided by the author.
**Lieb, 2014 asked about the existence of a general COI policy and/or a policy on interactions between medical students and industry.
I^2: Heterogeneity described by calculating I^2
